# A systematic review of cost-effectiveness, comparing traction to intramedullary nailing of femoral shaft fractures, in the less economically developed context

**DOI:** 10.1136/bmjgh-2017-000313

**Published:** 2017-09-25

**Authors:** Rebekah J Parkes, Gary Parkes, Kyle James

**Affiliations:** 1 School of Medicine and Dentistry, Queen Mary University of London, London, UK; 2 Department of Surgery, United Mission Hospital Tansen, Tansen, Nepal; 3 Department of Orthopaedics, Beit CURE International Malawi, Blantyre, Malawi

**Keywords:** Health economics < Health policies and all other topics, Orthopedic surgery

## Abstract

**Introduction:**

Femoral shaft fractures carry considerable morbidity and are increasingly common in less economically developed countries (LEDCs). Treatment options include traction and intramedullary (IM) nailing but in a limited-resource environment; cost-effectiveness is fundamental to policy development.

The objective herein was to evaluate the cost-effectiveness of moving from traction to IM nailing for femoral shaft fractures, in adults, in LEDCs. Incorporating a systematic review of complications and functional outcomes and a cost-minimization analysis.

**Methods:**

PubMed, EMBASE, Africa Journals Online and the Cochrane Library were searched from inception using the terms: femur* AND fracture AND traction AND (sign OR nail* OR intramedullary) AND (cost-effectiveness OR cost* OR outcome OR function) NOT paed* NOT child* NOT elastic NOT neck NOT intertrochanteric NOT periprosthetic (where asterisks indicate an unlimited truncation strategy). Abstracts were reviewed for all titles returned and full texts obtained as indicated. References of all relevant papers were also examined for further studies.

**Results:**

IM nailing has been successfully used in several institutions and reported infection, union and reoperation rates are encouraging, although no randomised control trials were identified. Three studies assessed the cost aspect and all found IM nailing to be the cheaper strategy.

**Conclusion:**

To date, the improved complication profile and reduced cost of treatment suggest that IM nailing is more cost-effective than traction. Evidence, however, is limited and the necessity for appropriate training and audit with the introduction of new techniques must be emphasised.

**Key questions**

**What is already known about this topic?**

- Femoral shaft fractures are increasingly common in less economically developed countries (LEDCs).
- Although many affluent counties use intramedullary (IM) nailing as a first-line treatment traction remains the primary management strategy in LEDCs.

**What are the new findings?**

- The mean infection rate across the cohort studies of IM nailing discussed herein was 3% compared with 27% in those managed with traction.
- The mean length of inpatient stay is longer in those managed with traction (54 days) versus (28 days) for IM fixation.
- Evidence is limited but suggests cost-effectiveness of moving from traction to IM nailing for both improved outcome and reduced cost.

**How might this influence practice?**

- Service planners considering transition from traction to operative management of femoral fractures should be encouraged but caution in the form of adequate training and monitoring must be emphasised.

## Introduction

In affluent countries, intramedullary (IM) nailing has been well established as the primary management strategy for femoral shaft fractures in mainstream orthopaedics for almost half a century. However, this is not the case worldwide and many hospitals in Less Economically Developed Countries (LEDCs) continue to implement traction as the mainstay of treatment. In their recent review, Kramer *et al* highlight the high incidence of complications and extended treatment period traction entails.^1^

As the rate of road traffic accidents in LEDCs rises, the management of femoral fractures which, represent 7.5% of resultant non-fatal injuries, is becoming a significant public health issue.^2 3^ In the context of limited resources, economic evaluation of the merits of competing therapy options is imperative to inform policy makers’ decisions regarding provision of care.

To be considered cost-effective, an intervention must be cheaper and more effective than the alternative or the increase in cost be outweighed by the benefit or the cost reduced substantially more than the quality of the outcome.^4^ Therefore, studies assessing various outcome measures and those evaluating costs will be considered.

In addition, logistics must also be considered, such as the lack of a C-arm or a reliable electrical source from which to run one in many institutions.^3^ This aspect has been recognised by the surgical implant generation network (SIGN) who have designed a solid locking IM nail that can be inserted without the need for a traction table or intraoperative imaging.^5^ The use of this implant is therefore central to many of the studies discussed herein.

For the purpose of this study, the term LEDCs equates to the countries referred to by the United Nations (UN) as ‘developing economies’.^6^ These tend to be dev­eloping countries but do not all feature among the least developed countries subgroup.^6 7^

## Aims

1. To compare outcomes in terms of complications (eg, infection and non-union) and function (eg, return to work).
2. To evaluate which method of femoral shaft fracture treatment (traction or IM nailing) is more expensive in LEDCs on a purely hospital stay resource-use basis (cost-minimisation analysis).
3. To combine these and consider the overall cost-effectiveness of moving from traditional traction to IM nailing.

## Research question

In the resource poor environment typical of LEDCs, is the initial outlay of investing in IM nailing for femoral shaft fractures worthwhile from a health economics perspective, in terms of overall treatment cost and health outcome?

It is hypothesised that although the cost of bed days is considerably lower in LEDCs, the early mobilisation associated with IM nailing is worthwhile enough to pursue.

Population:

- patients sustaining femoral shaft fractures.

Intervention:

- femoral nailing.

Comparison:

- traction.

Outcomes:

- absolute cost of treatment (however calculated);
- length of stay;
- complications including infection, malunion and non-union;
- function including timing and ability to mobilise and return to work.

Context:

- general hospitals in LEDCs.

## Method

This systematic review was conducted in accordance with the Preferred Reporting Items for Systematic Reviews and Meta-Analyses (PRISMA) guidelines.^8^

PubMed, EMBASE, Africa Journals Online and the Cochrane Library (from inception to present) were searched using the terms: femur* AND fracture AND traction AND (sign OR nail* OR intramedullary) AND (cost-effectiveness OR cost* OR outcome OR function) NOT paed* NOT child* NOT elastic NOT neck NOT intertrochanteric NOT periprosthetic (where asterisks indicate an unlimited truncation strategy). Two reviewers independently screened the result, applying the inclusion and exclusion criteria first to the title and abstract and then as necessary to the full text. Any discrepancy was resolved by discussion. The reference list of relevant papers was also examined for further studies in an attempt to minimise omissions.

Inclusion criteria:

- adult patients (≥16 years, skeletally mature or as defined by study);
- with a femoral shaft fracture;
- treating with IM nailing and/or traction;
- in a country listed on the UN developing economies or least developed countries list^6^;
- discussing an aspect of cost, outcome or function.

Exclusion criteria:

- paediatric patients;
- fractures of the femoral head or neck;
- patients with an ipsilateral acetabular fracture or lower limb amputation;
- periprosthetic and pathological fractures;
- cases using elastic nailing or external fixation;
- mixed long bone fracture cohorts, where figures for femoral fractures are not separately reported;
- descriptions of techniques or case reports;
- hospitals not functioning under restrictions in keeping with an LEDC setting.

The quality of economic studies was assessed using the Critical Appraisal Skills Programme (CASP) Economic Evaluation Checklist and data extracted and collated across all cohort studies for comparison including absolute cost of treatment, length of stay, complication rates, measures of function and time horizon.^9^ Where multiple traction methods are used, results for Perkins’ method were extracted.

## Results

**Figure 1:**
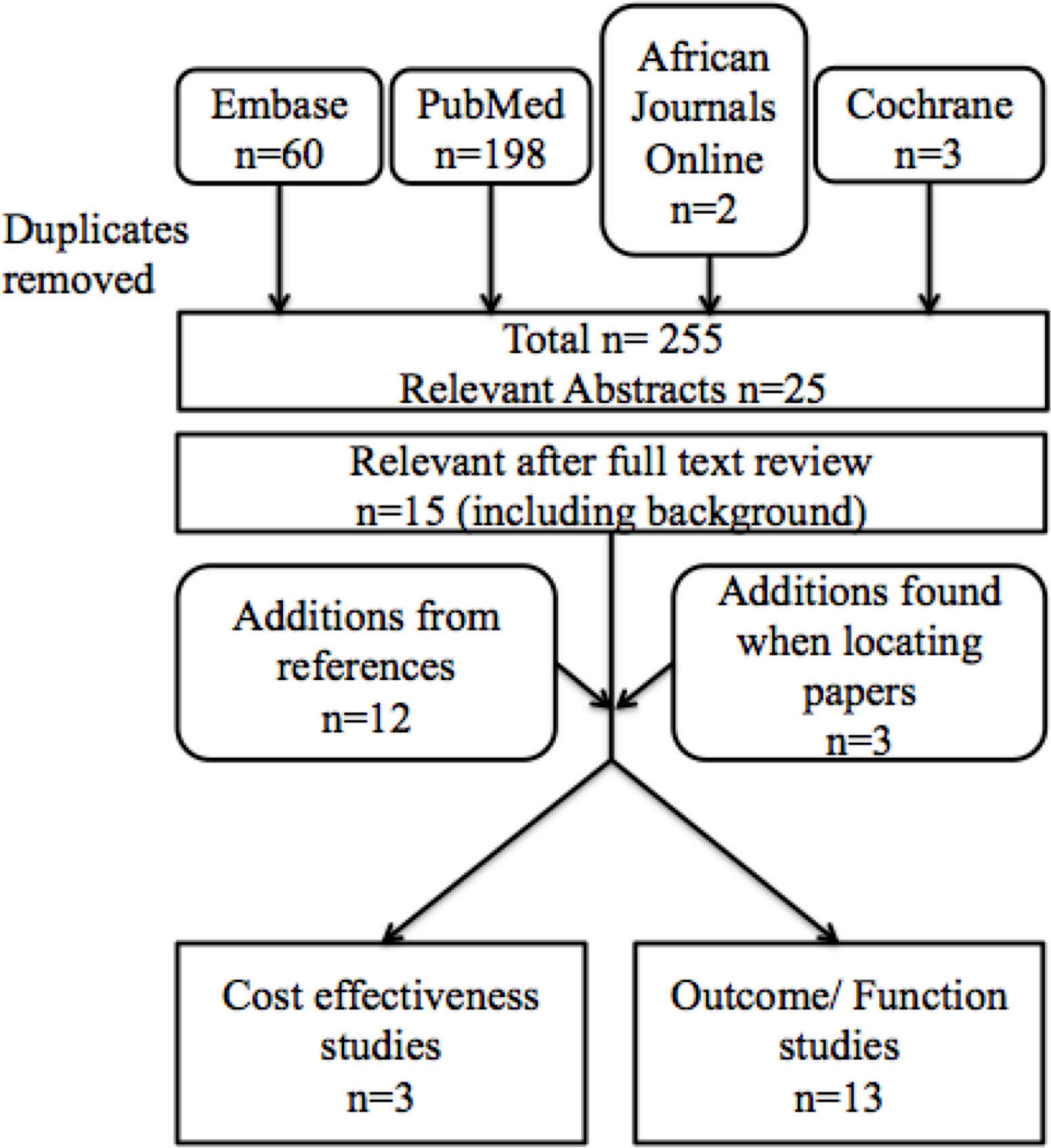
Flow diagram of study selection.

The search returned three cost-effectiveness studies and 13 cohort studies relating outcomes of traction and or IM fixation (figure 1). Only one of the cohort studies compared the two modalities. Fourteen other papers reported database analysis, qualitative aspects and background information, but no randomised control trials were evident. See online Supplementary file 1 for details of studies excluded during full text review.

10.1136/bmjgh-2017-000313.supp1 Supplementary file 1

Table 1 summarises the details of the included studies.

**Table 1:** Summary of effectiveness study characteristics

				Pearson 1977^10^	Usdin 1969^11^	Bewes 1974^12^	Mandrella 2002^13^	Gosselin & Lavaly 2007^14^	Boopalan *et al* 2014^15^	Aiyer *et al* 2006^16^	Soren *et al* 2009^17^	Sekempi *et al* 2011^18^	Chakraborty *et al* 2011^19^	Doorgakant & Mkandawire 2012^20^	Young *et al* 2013^21^	Bezabeh *et al* 2012^22^	Gosselin *et al* 2009^25^	Opondo *et al* 2013^26^	Kamau *et al* 2014^27^	Total	Mean
Country				Nigeria	South Africa	Tanzania	East Africa	Sierra Leone	India	India	Kenya	Uganda	Nepal	Malawi	Malawi	Ethiopia	Cambodia	Kenya	Kenya		
When				18 years 1954-1971	3 years	not reported	8 years 1991-1999	25 months 2003-2005	59 months 2003-2007	24 months year not stated	24 months 2005-2007	13 months 2007-2008	16 months 2010-2011	10 months 2010-2011	28 months 2010-2012	? 2007-2012	7 month recruitment 2007	12 months 2010-2011	6 months 2012-2013		
Age Included				20+	not reported	not reported	not reported	15+ skeletally mature	22+	20+	18+	15+	13+	14+	12+	16+	14+	19+	18+		
Mean Age				not reported	not reported	not reported	not reported	34.2	median 27	30	41 (includes tibia fractures)	31	not reported	34.5	33.3	approx 30	33	42	31.5		34
Gender		Male		not reported	not reported	not reported	not reported	43	15	160	45	37	not reported	13	114	60	not stated	115	45	647	81%
		Female		not reported	not reported	not reported	not reported	10	2	40	15	13	not reported	7	23	8	not stated	33	5	156	19%
Fracture age				recent but not explicitly stated	not reported	recent but not explicitly stated	not reported	<2 weeks	mean 13 weeks (4-44 weeks)	<14 days	41 recent	up to 33 days	recent but not explicitly stated	recent but not explicitly stated	32 delayed union	recent but not explicitly stated	<42 days		<7 days		
Type of study				retrospective cohort	cohort	cohort	cohort	retrospective cohort compared to another traction method	prospective cohort	retrospective cohort	prospective cohort	prospective cohort	retrospective cohort, much data presented jointly with tibial nailing	prospective cohort	prospective cohort	prospective cohort	retrospective cohort	quasi-experimental (patient choice)	prospective cohort		
Traction		No. patients		33	54	15	109	53	0	0	0	0	0	20	0	68	50	79	25	506	
		No. fractures		33	58	15	109	54	0	0	0	0	0	20	0	69	52	79	25	514	
IM nail		No. patients		0	67	0	0	0	17	200	60	50	6	0	137	0	37	69	25	668	
		No. fractures		0	67	0	0	0	17	200	60	50	6	0	141	0	37	69	25	672	
Open		traction		not reported	not reported	not reported	44 (40%)	4 (7.6%)	N/A	N/A	N/A	N/A	N/A	0	N/A	25 (36%)	16 (32%)	not reported	0		31%
		IM nailing		N/A	not reported	N/A	N/A	N/A	no (not explicit)	0	3 (5%)	0	0	N/A	7 (7%)	N/A	7 (19%)	not reported	0		7%
Follow-up		mean		"if they lived in the vicinity"		"very short"	"poor"	4.6 months	33 months	10 months				until inpatient discharge	381 days	8 months	6 months	12 weeks	3 months		
		range						3-9 months	6-72 months	6-12 months	some beyond 30 weeks	minimum 6 months/fracture healed				4-21 months	4+ months		3 months-119 days		
		percentage						67% reported on	100%		69% (includes tibial; minimum 58%)	71% reported on			79%						
Associated injuries deemed significant				no	not reported	not reported	some, level not reported	47.2%	53%	no (not explicit)	not reported	40% (excluded if major)	not reported	no	25%	not reported	no	100%	no		
Time to surgery		mean									13.2 days			17 days (SD 10) in acute		20.7 days					
		range										0-33 days							<7 days inclusion criteria		
Bed days		Traction	mean	65	49			52.1						46		45	52.3	60	66.7		54
			range	45-121				25-108						29-74		30-60+			44-119 days		
		IM nail	mean		38.5				7		10 (includes tibial)	20.1 (6.9 post op)			30 in acute		34.9	30	11.48		28 (excluding Soren et al.)
			range						4-20 days		3-22 (includes tibial)	2-11 post op			1 to 90				4-19 days		
Blood loss				no transfusions					5 required transfusion up to 2 pints	50-100ml					mean 279ml (SD202) fresh		mean 2.5 units transfused				
Infection	Traction	Pinsite	n	"common"			2 deep infections	23						7		8	14		excluded	52	27%
			percentage	one excluded patient died of related sepsis			rates pinsite infection not reported	43%						35% (41% of those with skeletal traction)		12%	27%				
	IM nailing	Deep	n						0	0	1	1	0		7		1		excluded	10	2%
			percentage						0%	0%	2%	2%	0%		5%		3%				
		Superficial	n						0	0	1	1	0		1		1		excluded	4	1%
			percentage						0%	0%	2%	2%	0%		1%		3%				
		Total	n						0	0	2	2	0		8		2		excluded	14	3%
			percentage						0%	0%	3%	4%	0%		6%		5%				
Union	Non-union	Traction	n	0	2	0	0	4								1	10			17	4%
			percentage	0%	3%	0%	0%	7%								1%	19%				
		IM nail	n						0	0		0			1		3			4	1%
			percentage						0%	0%		0%			1%		8%				
	Delayed Union	Traction	n				1							2					4	7	5%
			percentage				1%							10%					16%		
		IM nail	n						1	0		2							1	4	1%
			percentage						6%	0%		4%							4%		
	Malunion	Traction	n				0	5						1		1	2	34	9	52	13%
			percentage				0%	9%						5%		1%	4%	43%	36%		
		IM nail	n						0	0		4					0	21		25	7%
			percentage						0%	0%		8%					0%	30%			
	Shortening	Traction	n				0	3 (>2.5 cm)						2 (≥3cm)		11 (>2cm)				16	6%
			percentage				0%	6%						10%		16%					
		IM nail	n						1 (3.5cm)	0		0								1	0.4%
			percentage						6%	0%		0%									
	Refracture	Traction	n		2		5	2												9	4%
			percentage		3%		5%	4%													
		IM nail	n						0	0	1	2 (intra op)								3	1%
			percentage						0%	0%	2%	4%									
Futher theatre trips	Traction		n		6			2									2			10	6%
			percentage		10%			4%									4%				
	If all non union included		n	0	6		0	6								1	12			25	7%
			percentage	0%	10%		0%	11%								1%	23%				
	IM nail		n						3	0	3	4	1		9		3			23	5%
			percentage						18%	0%	5%	8%	17%		6%		5%				
Death			n		6										5						
Details				skin traction used predominantly, physio late introduction only	2 deaths in perkins group and 4 in kuntscher nailing. 7 "failures" in IM nail group	2 patients treated with kuntscher nail as soft tissue interposition suspected on x-ray	no deep vein thromboses, 41 cases gunshot wounds, complications clinically defined	4 pins also had to be resited for infection	20 weeks post injury and short pre op; image intensifier used	some intra operative x-ray used so only 8 opened at fracture site	4 screw loosening	2 pulmonary emboli pre-op	gapping at fracture site requiring dynamization	variable traction methods used, malunion case was one of two with shortening	32 delayed presenta­tions, all had intra­operative antibiotics, mobilisa­tion delayed as had to pay for crutches to be made		inpatient discharge only after suture removal 14/7 post op, 1/52 oral antibiotics routine in operative group		longest stay related to time to clear bills		

The earliest cohort study, 1954–1971, involved treatment with traction in Nigeria. The study includes paediatric patients; however, the format of data presentation allows extraction of findings for 33 patients, 20 years of age and over. Of these, only 11 had skeletal traction and although they do not provide figures for pin site infection, they indicate that it was common, even stating that one patient, excluded from analysis, died from pin site related septicaemia. Their focus is on duration of inpatient treatment, and aside from the absence of any cases of non-union or readmission, they do not comment on complications and follow-up patterns are unclear. Surprisingly, their mean of 65 inpatient bed days is only marginally higher than in later studies despite physiotherapy only being available during 2 years of the study, perhaps because multiply injured patients were excluded.^10^

Another study over 3 years during this period, in South Africa, compared cohorts of 54 patients treated with Perkins’ method, 67 with Küntscher nailing and a further group treated in Thomas’ splints. No detail is provided on how the method of treatment was selected. They highlight that pin site related ‘sepsis’ was seen only with Steinmann’s and not Denham’s pins, although there is no detail of diagnostic criteria. The mean hospital stay in the IM nailing group was as protracted as 5.5 weeks (seven for traction); however, there is a suggestion of crossover into this group when clinicians became frustrated with slow progress. The prevalence of severe injuries in the cohort, suggested by the mortality rate of 3.9%–11.8% across the groups, may also have delayed rehabilitation.^11^

Bewes in his 1974 series of 15 patients treated with Perkin’s traction in Tanzania focused largely on treatment methodology and subjective comparisons to other forms of traction. He reported clinical union in all cases but indicates that two patients were managed with IM nailing due to tissue interposition; cases that might have presented greater challenge to union.^12^

The fourth cohort, 1991–1999, was of 109 patients across Médecins Sans Frontières and Red Cross hospitals in East Africa receiving Perkins’ traction. In this study, there were a high proportion of open injuries (40%), the majority caused by ballistics. Amazingly, after appropriate debridement and wound management, only two patients developed deep infections. Again they allude to the presence of pin site infection but do not attempt to quantify this. The other complications reported are five refractures from mobilising too quickly and one delayed union. However, there was poor (not quantified) follow-up and complications were based on clinical assessment alone.^13^

The remaining studies cover shorter time intervals.

The next study, 2003–2005, of 41 patients in Sierra Leone had a much lower proportion of open fractures (7.6%); however, 47.2% had other significant injuries. Here, a minimum of 3 months follow-up was required for inclusion so some complications may represent overestimates, the assumption being that someone without problems would be less likely to return. Pin site infection was a major issue in this study with 42.6% of patients affected despite the relatively strict diagnostic criteria of loosening, purulent discharge or X-ray evidence. Two patients required operative intervention for sequestrum.^14^ Despite wider use of X-ray, a similar rate of refracture was seen as in the older East Africa study; however, the mean number of inpatient days was lower (52 compared with 65).^13 14^

A study focusing on neglected femoral fractures, 2003–2007 in India reviewed 17 cases of IM nailing. Unlike many of the other studies, an image intensifier was available. Nevertheless, the study was included as the delay in treatment is in keeping with an LEDC healthcare environment. Given the extent of the initial deformities, a single case of shortening and the requirement for blood transfusion (29%) are relatively acceptable. Further operations were for thrombectomy and locking screw adjustment/removal. The authors report that all patients were able to return to work.^15^

Another group in India studied a much larger cohort of 200 patients with fresh fractures (<14 days old), recording blood loss of only 50–100 mL. However, although they did not have the instantaneous feedback of a C-arm, they did have some access to intraoperative X-ray meaning a large percentage of cases could be completed with a closed technique (all except eight). Along with an absence of open fractures, this access to imaging goes some way to explaining the total absence of infective complications in these two studies.^16^

A cohort of 60 femoral fractures, in a provincial hospital in Kenya, was managed with SIGN nailing. Forty-one of these were fresh fractures but the study also includes cases where complications were being managed as well as an additional 20 patients with tibial nailings; not all outcomes are reported separately clouding the picture. However, the infective complication rate appears to be low (3% in femoral cases) and postoperative length of stay was a mean of 10 days across the cohort, despite a high proportion of open fractures among the tibial group. Four cases of screw loosening are noted but this is not expanded on and may represent further cases of infection.^17^

Fifty patients in Uganda were treated with SIGN nailing of closed femoral fractures and followed for a minimum of 6 months or until their fracture was healed. Another 20 were excluded for inadequate follow-up, which may bias results as mentioned previously. The results suggest technical difficulties with two intraoperative patella fractures (attributed to knee stiffness in cases of delayed retrograde nailing), four malunions (>10 degrees coronal plane) and two delayed unions.^18^ These issues could be related to individuals’ learning curves, fracture configuration or timing of treatment.

A small mixed cohort of 16 tibia and six femur fractures in Nepal evaluated the introduction of SIGN nailing. One case of over distraction in a femur required dynamisation.^19^

One of the more recent cohorts of traction (2010–2011) reviewed 20 patients in Malawi. The treatment in this group was heterogeneous with both skin traction and Braun frame methods employed. Seven of the 17 patients with skeletal traction required oral antibiotics for pin site infections, giving a rate similar to other series.^20^

Another study from Malawi focuses on IM nailing in 137 patients (77% fresh fractures). This group went to great lengths to maximise follow-up rates and found no infections in the patients that had not attended for follow-up. However, they still saw one of the highest infection rates 6% (5% deep) and on statistical testing similar rates independent of HIV status. They also highlight areas of inefficiency in terms of mean 17 (SD 10) bed days preoperatively and delay in mobilisation postoperatively ‘for the carpenter to make their crutches’.^21^

The final cohort of patients treated with Perkins’ traction in Ethiopia consists of 68 cases. They saw pin site infection in only 12% of cases despite 36% being open fractures. Sixteen per cent of patients had leg-length discrepancy; however, this may be largely explained by their using the strictest definition across the studies (>2 cm).^22^

Young *et al* reviewed the SIGN database looking at infection rates in 2010 and revisited it the following year. They found that follow-up rates above 5% did not increase the infection rate seen.^23^ If rates were based only on patients attending follow-up, 3.2% (95% CI 2.9 to 3.5) had infection, dropping to 0.8% (95% CI 0.7 to 0.9) against the total number of those treated (slightly higher percentages are seen in the least economically developed countries).^24^ The first figure is similar to the mean infection rate across the cohort studies of IM nailing discussed herein (3%); however, prophylactic antibiotics, which can reduce the relative risk of infection by as much as 29%, are rarely mentioned.^24^

The overall infection rate is higher in those cohorts treated with traction (27%) as is the need for further surgery (7% vs 5%) and the mean length of stay is longer (54 vs 28 days), although some bias is suggested by higher open fracture rates.

### Economic Studies

The first economic study was of 97 patients in 2007 in Cambodia on the introduction of the SIGN nail to their hospital. The study sought to evaluate the change in cost and outcome associated with the introduction of IM nailing in a hospital run by a non-government organisation (NGO) and collected data retrospectively. The time horizon although not specifically defined was approximately 6 months.

They saw the mean price of treatment drop from US$941 to 820 with fewer bed days and higher rates of union.^25^ However, the subgroup delineation was not pure, with a number of patients receiving a period of traction before undergoing fixation (mean 20.7 days). Complications were reported in natural units to allow comparison of effectiveness but quality of life measures were not available. Costs were clearly displayed from a payer/provider perspective and there was evidence of incremental cost analysis, for instance, in estimates around the cost of later intervention for non-unions. The paper describes a change in the profile of complications encountered and concludes that better outcome was achieved at lower expenditure with IM nailing. Variation in cost, particularly with contamination of the operative group with periods of traction likely lengthening stay, was considered and if deducted would strengthen the argument for change.^25^

The other two economic studies were performed in larger hospitals in Kenya including 148 and 50 patients, respectively. Opondo *et al* prospectively studied care in a Provincial Hospital in 2010. They aimed to evaluate the difference in costs (hospital bills) and consequences based on patient choice of whether to be treated with traction or IM nailing. Outcomes were reported in terms of bed days, ability to mobilise unaided and ‘complications’. However, there is a disconcerting lack of detail about ‘complications’, for example, what they were, what defines them as local or systemic or indeed whether they were self-limiting or required costly intervention. Overall, there was a reduction in price from US$167 to 120. However, the components included in the economic calculations are listed but only the total cost is presented and no breakdown given. Therefore, neither incremental nor sensitivity analysis are evident. They found that IM nailing was the cheaper option with no significant change in ‘complications’ and improved ability to mobilise at 12 weeks (the time horizon).^26^

The final study performed by Kamau *et al* was undertaken in 2012/2013 in the National Hospital, Kenya. They reviewed cost as compared with fracture union from the perspective of hospital cost with a 3-month (or hospital discharge if later) time horizon. To qualify in the operative group, patients had to have their procedure within 1 week, clearly defining the study groups. Effectiveness was viewed from the attainment of union.^27^ The record of effects was somewhat limited, with a time horizon not long enough to evaluate for non-union and no comment was made on complications. Contacting the author revealed that only uncomplicated cases were evaluated, with all cases of infection excluded (DM Kamau, personal communication, 2016). This is understandable in a small study where such cases could dramatically skew results. However, this means that opportunity costs related to the complications of nailing do not form part of the analysis. The costs detailed do incorporate many elements and the relative weightings of these contributors are discussed. They found that routine management with IM nailing in uncomplicated cases is cheaper than traction (US$798 compared with US$640) and is associated with higher union rates.^27^

Overall, reduction in price was seen in all studies but in monetary terms the absolute values were very variable.

Table 2 summarises the comparative prices across the economic studies.

**Table 2:** Collated costing and estimated comparative cost breakdown for economic studies

		Gosselin et al. 2009^25^		Kamau et al. 2014^27^				Opondo et al. 2013^26^			
		Cambodia		Kenya				Kenya			
		Costs is US$		Costs is Kshs		Costs in US$ (exchange rate 1 US$=85 Kshs)		Costs is Kshs		Cost in US$ (exchange rate 1 US$=81.484 Kshs)	
		Traction	Nailing	Traction	Nailing	Traction	Nailing	Traction	Nailing	Traction	Nailing
No. patients		50	37	25	25	25	25	79	69	79	69
Traction	Pins	156	24								
	Temporary traction	0	60								
	Perkin’s frame	250	40								
	Crutches	750	555								
	Cast-brace	2640	0								
	Total	3796	679	49149	0	579	0				
	Per patient	76	18	1966	0	23	0				
	Percentage	8.1%	2.2%			2.9%	0.0%				
Rehab and monitoring monitoring	X-rays	442	189								
	Physiotherapy	2150	1113								
	Total	2592	1302	396583	107401	4668	1264				
	Per patient	52	35	15863	4296	187	51				
	Percentage	5.5%	4.3%			23.4%	7.9%				
Accommodation	Pre-op		11872								
	Post-op		8143								
	Total	40532	20015	1249068	228398	14703	2688				
	Per patient	811	541	49963	9136	588	108				
	Percentage	86.1%	66.0%			73.7%	16.8%				
Theatre	Implant	0	5550								
	Theatre time	0	2082								
	Blood	0	460								
	Total	0	8092	0	1023712	0	12048				
	Per patient	0	219	0	40948	0	482				
	Percentage	0%	26.7%			0%	75.3%				
Miscellaneousous	Other adjuncts	140	259								
Total		47060	30347	1694800	1359511	19950	16000	1073926	673509	13180	8266
		47060	30347	67792	54380	798	640				
	Per patient	941	820	67792	54380	798	640	13594	9761	167	120
	Per fracture	905	820								

## Discussion

The standard management of femoral shaft fractures in LEDCs is that of traction and the studies collated herein demonstrate the success of this treatment with a mean non-union rate of only 4%, delayed union of 5% and refracture of 4%. However, malunion is seen in 13% with significant shortening in 6% which may have significant implication for the functional part these individuals are able to play in society. Further, some of these patients may require delayed operative intervention with resource implications. There is little in the literature to indicate how many of these individuals seek such further treatment.

The main difficulty with traction, however, is the longevity of treatment (mean 54 inpatient days) which will inevitably have negative economic impact on many patients and in many cases will result in another family member also being removed from the work force as they provide personal care at the hospital. The studies here see this reduced to a mean of 28 days with IM nailing. There is likely to be scope for reducing this period further as some of the studies from which this mean is derived has considerable lead time before surgery was undertaken (however, various barriers may exist to expedient surgery).

The critical area that has traditionally resulted in IM nailing being avoided is fear over infection in the LEDC context but the results here are very reassuring with only 3% having infection compared with 27% receiving traction. However, the reporting of infection for the two modalities is from different studies so direct comparison should be cautious as the necessary surgical management of deep infection will vary.

In 1973, Carr and Wingo published a study of the cost-effectiveness of IM nailing as compared with traction in the USA. Despite a few inconsistencies seen among the figures for complications presented in their paper, they showed relatively convincingly that IM nailing as compared with traction resulted in reduced hospital stay and earlier return to work. They modelled cost of treatment, projecting forward 5 years and noted that IM nailing cost 20%–30% less than traction with the difference expected to increase. They noted that the cost of treatments and their adjuncts were growing in cost more quickly than that of accommodation. Breakdowns of this calculation are not provided, so it is not possible to define which aspects contributed most to this projection (for instance, it is possible that the cost of spica casting in the traction population added disproportionately to the overall expense). However, the USA’s health system sits in stark contrast to that of many LEDCs so the benefit seen there may not be transferable.^28^

Some decades on, the first economic analysis performed on this topic in an LEDC was by Gosselin *et al* in 2007, in a hospital run by an NGO, in Cambodia. A further two economic studies later followed in larger hospitals in Kenya. In all cases, similarly to the earlier USA-based paper traction rather than no treatment is used as the baseline with it widely viewed that outcomes are not acceptable in the majority of cases where no intervention is made. All three studies noted a reduction in price associated with a reduction in bed days. In the studies that provided cost breakdown, this is demonstrated in a reduction in accommodation cost which forms a large percentage of the overall price of treatment.

All the economic studies were limited in justifying effectiveness from the literature and largely relied on their own outcomes to define this. None of the researchers employed discounting (techniques in economics for adjustment based on the concept that society values early reward over delayed gratification), but this is not unreasonable given their short time horizons. The context of these hospitals is appropriate and they do fulfil the inclusion criteria set out here. However, by nature of being centres of research and higher level institutions or in one case an NGO hospital supported by an affluent country, these hospitals will likely be better resourced, with better trained staff than many other centres in LEDCs. Indeed, the very fact that SIGN nails are used in these institutions means that they have satisfied the charity of their experience and follow-up abilities, which other groups may struggle to demonstrate.

Regarding resources, all three groups included the cost of implants in the calculations for the IM group although this aspect is only presented in the Cambodian study. Kamau *et al* used three kinds of IM nail, SIGN (supplied free of charge), Küntscher nails and occasionally TREU nails, which complicates the cost profile (DM Kamau, personal communication, 2016). With theatre costs, including that of the implant, making up 75.3% of the overall treatment cost in this study, which implant was used is likely to have a significant impact on the cost-minimisation calculations. In the study by Opondo *et al*, the costs are very different from those in Kamau *et al*’s study despite being conducted in the same country, only a year apart. There are some anomalies in the data in Opondo’s study with one bill as low as 400Ksh which equates to about US$5 in the conservative group and the mean cost in the operative group is approximately US$120 (which the author informs me includes the price of the nail) (E Opondo, personal communication, 2016), while the cost of providing a nail is US$150 according to SIGN (J St John, personal communication, 2016). It is unclear whether this is a miscalculation or rather represents a degree of subsidisation, as if you charge the patient in your hospital SIGN charge for the nail, while if care is provided for free they do not charge for implants.

Considering equipment expense, one group compared the cost of sourcing traction pins for Malawi, Kenya and Tanzania, finding local manufacture to be significantly cheaper (p<0.001).^29^ A laudable attempt at simple innovation. However, such saving would have only minimal impact on overall cost of traction treatment, with traction pins making up 0.33% of the cost in the Cambodian study and the entirety of the traction assembly contributing only 2.9% of costs in Kamau *et al*’s paper.^25 26^ Also the authors acknowledge, using local metal fabrication shops, appropriate quality for clinical use cannot be guaranteed making the ethical position of pursuing this idea complex.^29^ This is an issue that does not affect the current manufacture process for SIGN nails as they are produced under an internationally approved standard.^5^

None of the cost-effectiveness studies have attempted to evaluate costs outside of direct treatment, for example, loss of earnings for the patient and carers. This is a difficult area to study with patients coming from different backgrounds, with high levels of subsistence and informal employment in many regions. One qualitative study in Uganda of a mixed cohort of 35 patients with tibial and femoral fractures has attempted to explore the issues. They found the effects wide ranging, with patients supporting a mean of 5.7 dependants (and anxious about ongoing ability to do so), delaying treatment due to prohibitive travel costs and children being taken out of school due to lack of finance.^30^ This highlights access issues regarding how broadly a policy of IM nailing can be rolled out, especially as not all hospitals have ready access to orthopaedic surgeons.^2^ However, SIGN have been strategic in establishing their technique through maintaining device simplicity, allowing hospitals to become regional training centres and providing a supply line integrated with cases reporting, affording a degree of quality assurance.^3 5^

### Limitations

None of the studies described herein are randomised control trials, rather they are highly heterogeneous and many only consider one of the therapeutic methods under discussion. Furthermore, the majority summarise data as means in the absence of a full data set or SD making statistical comparison and combination unreliable.

Knee stiffness has not been considered among the outcomes as it is variably reported and success is largely the product of the quality of physiotherapy received (which is difficult to evaluate). Also physiotherapy is the area that has subjectively seen the most improvement across the time spanned by the studies further complicating comparison (Gosselin RA. 2016, personal communication, January 23).

Aside from the one qualitative paper discussed above, there is a noticeable silence in the literature regarding the wider impact of treatment in terms of the functional (eg, return to work) and societal (eg, loss of children’s education) outcomes.

The economic studies are small observational cohorts, and the focus is on a relatively simplistic price in the form of hospital fees (payee interface) rather than true cost of the treatments, with short time horizons and no information regarding wider social impact or patients’ later contribution to society (eg, though return to work).

In line with the concept of discounting, it is also unknown how willing patients are to pay for a one-off surgical intervention in comparison to a longer hospital stay and whether this will vary between cultures.

## Conclusion

The studies discussed herein suggest that a transition from traction methods to IM nailing for femoral shaft fractures is beneficial in terms of union, hospital stay and cost-minimisation. Therefore, this would appear to be a cost-effective strategy. However, the evidence is limited and the necessity for appropriate training and audit with the introduction of new techniques must be emphasised. In regions where the logistical barriers to IM nailing are ongoing (eg, absence of a surgeon), traction should remain the modus operandi.
