# Supplementary material for: A systematic review of cost-effectiveness, comparing traction to intramedullary nailing of femoral shaft fractures, in the less economically developed context
Source: BMJ Glob Health. 2017 Sep 25;2(3):e000313. doi: 10.1136/bmjgh-2017-000313 (PMC5623315; doi:10.1136/bmjgh-2017-000313)
Supplement: Supplementary file 1 [file bmjgh-2017-000313supp001.pdf]

Appendices

Appendix 1

Table showing excluded cohort studies with reason for elimination

Study	Type	Reason for exclusion
Buxton 1981(31)	Retrospective cohort	Performed in England (not an LEDC).
Carr & Wingo 1973 (28)	Economic analysis	Performed in United States of America (not an LEDC).
Churchill-Davidson 1966 (32)	Cohort study	Performed in England (not an LEDC) and includes pathological fractures.
Clasper & Rowley 2009 (33)	Retrospective cohort	Exclusively ballistics injuries<br>Included femoral neck fractures<br>Conservative method not explicitly defined
Gurkan et al. 2009 (34)	Cohort study	Hospital not in keeping with typical low/ middle-income country e.g. access to MRI.
Ifesanya & Alonge 2012 (35)	Retrospective cohort	Mixed, predominantly tibial cohort without breakdown.
Ikem et al. 2001(36)	Prospective cohort	Mixed cohort predominantly tibia fractures managed with traction and external fixation. Outcomes are not reported by management strategy.
Ikem et al. 2007 (37)	Prospective cohort	Mixed cohort of long bone fractures, only 65% femurs and no breakdown of outcomes.
Ikpeme et al. 2011 (38)	Prospective cohort	Mixed cohort of tibia and femoral fractures undergoing IM nailing but results not reported independently.
Malik et al. 2004 (39)	Retrospective cohort	Performed in England (not an LEDC).
Miller et al. 1978 (40)	Retrospective cohort	Performed in USA (not an LEDC).

Patel 2014 (41)	Retrospective cohort	Performed in US Level 1 Academic Trauma Centre (not a LEDC). [Noted from abstract]
Procter 1962 (42)	Cohort study	Included pathological fractures
Rowntree & Getty 1981 (43)	Cohort study	Performed in England (not an LEDC).
Strecker et al. 1994 (44)	Retrospective cohort	Only 8 cases of IM fixation among 123 trauma patients discussed. The wider study group included paediatric patients and patient characteristics in this small subgroup were not defined. Also of note neither reamed nor locking techniques were applied and the only complication separately reported was a single non-union.
Yu et al. 2014 (45)	Cohort study	China is a developing economy but the study is conducted in a military institution and not representative of true LEDC situation. [Noted from abstract].
Zain Elabdien et al. 1984 (46)	Cohort study	Performed in Sweden (not an LEDC) and focuses on Ender nails (elastic nailing technique).

Appendix 2

Summary Table of Cost-effectiveness studies analysis using the CASP (Critical Appraisal Skills Programme) Economic Evaluation Tool

	Cambodia: Gosselin et al. 2009	Kenya: Kamau et al. 2014	Kenya: Opondo et al. 2013
1.Was a well-defined question posed?	☑Yes The purpose of the paper was to assess both cost and change in outcome at the introduction of SIGN nailing, with a time horizon of approximately 6 months.	☑Yes To assess cost and consequences of IM nailing verses traction in uncomplicated cases (focusing on union), with a 3-month (or discharge if later) time horizon.	☑Yes To assess cost and consequences (complications not broken down) of IM nailing verses traction from the perspective of hospital bills and a time horizon of 12 weeks.
2. Was a comprehensive description of the competing alternatives given?	☑Can't Tell The decision tree was not strictly adhered due to delays that resulted in some patients having traction before nailing.	☑Yes If operated on within one week then patients could be included in the operative group.	☑Yes Patient decision.
3.Does the paper provide evidence that the programme would be effective?	☑Yes- (weak) Reports complications but there are no RCTs or systematic reviews to judge effectiveness.	☑Yes- (weak) Reports on union rates but there are no RCTs or systematic reviews to judge effectiveness.	☑Can't tell Reports some 'complications' but there are no RCTs or systematic reviews

			to judge effectiveness.
4. Were the effects of the intervention identified, measured and valued appropriately?	☑Yes<br>Natural units reported.	☑Yes<br>Natural units but length of follow-up does not allow identification of non-unions and no other complications are considered.	☑Yes<br>Natural units but details of 'complications' not discussed.
5. Were all important and relevant resources required and health outcome costs for each alternative identified, measured in appropriate units and valued credibly?	☑Yes<br>Analysed from payer provider perspective, how the calculations are derived is clearly demonstrated and calculations for the management of non-union are also discussed.	☑No<br>Costs detailed and many elements considered but opportunity costs associated with complications were not evaluated.	☑Can't tell<br>Components listed but values not given, only totals stated.
6. Were costs and consequences adjusted for different times at which they occurred?	☑No<br>Discounting was not applied but is not really relevant given perspective and short time horizon.	☑No<br>Discounting was not applied but is not really relevant given perspective and short time horizon.	☑No<br>Discounting was not applied but is not really relevant given perspective and short time horizon.
7. What were the results of the evaluation?	There is an altered profile of complications but	Management with IM nailing in the absence of	No significant change in 'complications',

	overall it is cheaper to perform IM nailing.	complications is cheaper than traction and associated with higher union rates.	function at 12 weeks improved and IM nailing was cheaper than traction.
8. Was an incremental analysis of the consequences and cost of alternatives performed?	☑Yes<br>Breakdown was given.	☑Yes<br>Breakdown of biggest contributors to cost was given.	☑No<br>Not presented.
9. Was an adequate sensitivity analysis performed?	☑Yes<br>Variations in costs and differences were considered.	☑No<br>Not performed.	Only one overall cost presented.
10. Is the programme likely to be equally effective in your context or setting?	☑Yes<br>Appropriate setting, represents transition time.	☑Can't tell<br>National hospital (Level 6) so may not be directly transferable to majority of institutions.	☑Can't tell<br>Level 5 hospital so may not be directly transferable to majority of institutions.
11. Are the costs translatable to your setting?	☑Yes<br>Similar settings figures will likely vary a little between countries.	☑Yes<br>Similar settings figures will likely vary a little between countries.	☑Can't tell<br>Possible via reviewing exchange rates from the period. Similar settings figures will likely vary a little between countries.

12. Is it worth doing in your setting?	☑Yes	☑Yes	☑Yes

Additional References

31. Buxton RA. The use of Perkins' traction in the treatment of femoral shaft fractures. Bone Jt J. 1981 Aug 1;63-B(3):362–6.

32. Churchill-Davidson D. The Results of Early and Delayed Internal Fixation of Fractures of the Shaft of the Femur. Postgrad Med J. 1966 May;42(487):297-9.

33. Clasper JC, Rowley DI. Outcome, following significant delays in initial surgery, of ballistic femoral fractures managed without internal or external fixation. J Bone Joint Surg Br. 2009 Jan;91(1):97–101.

34.. Gurkan V, Orhun H, Doganay M, Salioğlu F, Ercan T, Dursun M, et al. Retrograde intramedullary interlocking nailing in fractures of the distal femur. Acta Orthop Traumatol Turc. 2009 Jul;43(3):199–205.

35. Ifesanya AO, Alonge TO. Operative stabilization of open long bone fractures: A tropical tertiary hospital experience. Niger Med J J Niger Med Assoc. 2012;53(1):16–20.

36. Ikem I, Oginni L, Bamgboye E. Open fractures of the lower limb in Nigeria. Int Orthop. 2001 Dec;25(6):386–8.

37. Ikem IC, Ogunlusi JD, Ine HR. Achieving interlocking nails without using an image intensifier. Int Orthop. 2007 Aug;31(4):487–90.

38. Ikpeme I, Ngim N, Udosen A, Onuba O, Enembe O, Bello S. External jig-aided intramedullary interlocking nailing of diaphyseal fractures: experience from a tropical developing centre. Int Orthop. 2011 Jan;35(1):107–11.

39. Malik MHA, Harwood P, Diggle P, Khan SA. Factors affecting rates of Infection and nonunion in intramedullary nailing. J Bone Joint Surg Br. 2004 May;86(4):556–60.

40. Miller C.W., Anderson L., Grossman J., Grant G. Comparison of three treatments for fractures of the diaphysis of the femur. Surg Gynecol Obstet. 1978;146(4):572–6.

41. Patel NMM, Yoon RS, Cantlon MB, Koerner JD, Donegan DJ, Liporace FA. Intramedullary Nailing of Diaphyseal Femur Fractures Secondary to Gunshot Wounds: Predictors of Postoperative Malrotation. J Orthop Trauma. 2014 Dec;28(12):711–4.

42. Procter DS. The functional treatment of fractures of the femur. South Afr Med J Suid-Afr Tydskr Vir Geneeskd. 1962 Jul 28;36:598–601.

43. Rowntree M., Getty C.J.M. The knee after midshaft femoral fracture treatment: A comparison of three methods. Injury. 1981;13(2):125–30.

44. Strecker W, Fleischmann W, Suger G, Schulte, M, Kinzl L. Grenzen der operativen Frakturbehandlung in tropischen Ländern. Tropenmed Parasitol. 1994;16:1–8.

45. Yu Z., Zhang K., Ren P., Zhang X., Chen J. Clinical evaluations of femoral shaft fractures treated with the retrograde interlocking intramedullary nail and close reduction. Int Med J. 2014;21(3):291–4.

46. Zain Elabdien B.S., Olerud S., Karlstrom G. Subtrochanteric fractures. Classification and results of Ender nailing. Arch Orthop Trauma Surg. 1984;103(4):241–50.
